# In Pulmonary Paracoccidioidomycosis IL-10 Deficiency Leads to Increased Immunity and Regressive Infection without Enhancing Tissue Pathology

**DOI:** 10.1371/journal.pntd.0002512

**Published:** 2013-10-24

**Authors:** Tânia A. Costa, Silvia B. Bazan, Claudia Feriotti, Eliseu F. Araújo, Ênio J. Bassi, Flávio V. Loures, Vera L. G. Calich

**Affiliations:** Departamento de Imunologia, Instituto de Ciências Biomédicas, Universidade de São Paulo, São Paulo, São Paulo, Brazil; University of California San Diego School of Medicine, United States of America

## Abstract

**Background:**

Cellular immunity is the main defense mechanism in paracoccidioidomycosis (PCM), the most important systemic mycosis in Latin America. Th1 immunity and IFN-γ activated macrophages are fundamental to immunoprotection that is antagonized by IL-10, an anti-inflammatory cytokine. Both in human and experimental PCM, several evidences indicate that the suppressive effect of IL-10 causes detrimental effects to infected hosts. Because direct studies have not been performed, this study was aimed to characterize the function of IL-10 in pulmonary PCM.

**Methodology/Principal Findings:**

Wild type (WT) and IL-10^−/−^ C57BL/6 mice were used to characterize the role of IL-10 in the innate and adaptive immunity against *Paracoccidioides brasiliensis* (Pb) infection. We verified that Pb-infected peritoneal macrophages from IL-10^−/−^ mice presented higher phagocytic and fungicidal activities than WT macrophages, and these activities were associated with elevated production of IFN-γ, TNF-α, nitric oxide (NO) and MCP-1. For in vivo studies, IL-10^−/−^ and WT mice were i.t. infected with 1×10^6^ Pb yeasts and studied at several post-infection periods. Compared to WT mice, IL-10^−/−^ mice showed increased resistance to *P. brasiliensis* infection as determined by the progressive control of pulmonary fungal loads and total clearance of fungal cells from dissemination organs. This behavior was accompanied by enhanced delayed-type hypersensitivity reactions, precocious humoral immunity and controlled tissue pathology resulting in increased survival times. In addition, IL-10^−/−^ mice developed precocious T cell immunity mediated by increased numbers of lung infiltrating effector/memory CD4^+^ and CD8^+^ T cells. The inflammatory reactions and the production of Th1/Th2/Th17 cytokines were reduced at late phases of infection, paralleling the regressive infection of IL-10^−/−^ mice.

**Conclusions/Significance:**

Our work demonstrates for the first time that IL-10 plays a detrimental effect to pulmonary PCM due to its suppressive effect on the innate and adaptive immunity resulting in progressive infection and precocious mortality of infected hosts.

## Author Summary

Paracoccidioidomycosis, the most important deep mycosis from Latin America, is acquired by inhalation of fungal spores. The pulmonary infection can remain as a quiescent infection or evolve to overt, life-threatening disease. Immunoprotection is mainly mediated by Th1 lymphocytes secreting IFN- γ, the most important macrophage activating cytokine. It is well established that the severe forms of infection are associated with elevated production of anti-inflammatory or suppressive cytokines such as IL-10. However, direct approaches investigating the role of this cytokine in pulmonary paracoccidioidomycosis were never employed. This led us to investigate the innate and adaptive aspects of immunity in pulmonary paracoccidioidomycosis using IL-10-deficient mice in comparison with their IL-10-normal counterparts. We verified that IL-10 absence leads to a regressive disease, resulting in reduced mortality rates of infected mice. This better disease outcome was associated with precocious and enhanced mechanisms of innate and adaptive immunity that allow the control of fungal growth without excessive inflammatory reactions and harmful tissue pathology. These evidences on the detrimental effects of IL-10 to pulmonary paracoccidioidomycosis suggest that therapeutic measures aimed to control IL-10 production or activity could exert a protective effect to this severe fungal pathology.

## Introduction

The clinical significance of fungal infections has increased dramatically in the past decades. Fungi are associated with a broad spectrum of diseases in humans, including self-limiting cutaneous or pulmonary infections to disseminated life-threatening diseases [1], [2]. It has been demonstrated that host resistance to fungal infections relies on the induction of cellular immunity, involving T cells, cytokines and effector phagocytes [1], [2]. While protection against fungal infections mainly requires the development of T helper (Th)-type of adaptive immunity, fungal susceptibility is mostly associated with the development of Th2-type responses or production of immunosuppressive cytokines, such as interleukin (IL)-10 [3]. More recently, Th17 cells have been associated with immunoprotection or excessive tissue pathology, whereas regulatory T cells (Treg) have been shown to play an essential role in the control of innate and adaptive immunity to fungal infections [4], [5].

Paracoccidioidomycosis (PCM), an important endemic deep mycosis in Latin America, is a chronic granulomatous disease caused by the dimorphic fungus *Paracoccidioides brasiliensis* [6]. In a murine model of pulmonary infection developed by our group, B10.A and A/J mice behaved as susceptible and resistant strains to *P. brasiliensis* infection, respectively. Similarly to the human disease, susceptibility was linked to depressed cellular immunity associated with enhanced IL-10 production and absence of IFN- γ synthesis [7], [8], [9]. In addition, in some experimental settings Th17 and Treg cells were shown to exert detrimental effects to pulmonary PCM. In the absence of TLR2 signaling, excessive inflammatory reactions were concomitant with increased Th17 expansion [4]. Furthermore, TGF-β- and IL-10-secreting Treg cells were associated with severe PCM due to their suppressive effect on the innate and adaptive immunity of resistant and susceptible mice [5].

IL-10, a regulatory cytokine, is known to be expressed by a variety of cells types including macrophages, dendritic cell (DC) subsets, B cells, neutrophils, eosinophils, mast cells, natural killer (NK) cells and several T-cell subpopulations (Th1, Th2, Th9, Th17, Treg) [10]–[16]. The anti-inflammatory properties of IL-10 are associated to its inhibitory activity on antigen-presenting cells (APCs) such as macrophages and DCs [17]. IL-10 has been shown to antagonize the expression of major histocompatibility complex class II (MHCII) proteins and co-stimulatory molecules (CD80/CD86), as well as the production and activity of pro-inflammatory cytokines (IL-1β, IL-6, IL-12, IL-18 and TNF-α) and chemokines (MCP1, MCP5, RANTES, IL-8, IP-10 and MIP-2). Thus, IL-10 can indirectly act on T-cell development and differentiation via the inhibition of macrophage/DC function [11], [14], [18]. Nonetheless, IL-10 can also directly affect T cells, down regulating the proliferation of and the cytokine synthesis by CD4^+^ T cells [15]. The potent inhibition of IL-12 production caused by IL-10, besides negatively affecting IFN-γ synthesis, prevents the development of Th1 immunity and favors the maintenance of a Th2 response [19], [20].

Importantly, recent studies have demonstrated that conventional Th1 and Th17 cells also secrete IL-10 that acts as a critical regulator of inflammatory reactions caused by these T cell subsets [21], [22].

Recent evidences linking impairment in the IL-12/interferon (IFN)-γ axis to susceptibility to disseminated PCM [23], [24], and the high levels of IL-10 detected in the severe forms of various mycosis [25]–[27], [1], [2], strongly indicate that IL-10 exerts deleterious effects on fungal infections. Moreover, in studies performed by Romano et al., peripheral blood mononuclear cell cultures from PCM patients, which present defective IL-12 and IFN-γ synthesis, showed an augmented expression of both cytokines upon IL-10 neutralization [28], [29]. In the murine model of PCM, IL-10 was also the prominent and early Th2 cytokine observed after antigen stimulation of lymph node cells from susceptible mice whereas resistant mice presented a delayed secretion of this cytokine [7], [30]. In addition, IL-10 has been found to suppress the IFN-γ-induced production of nitric oxide (NO), decreasing the phagocytic and fungicidal activity of human and mice phagocytes against pathogenic fungi, including *P. brasiliensis* [31], [32]. Such observations have been corroborated by studies using IL-10-specific monoclonal antibody or IL-10^−/−^ knockout mice, which showed increased resistance to distinct fungal pathogens, such as *Aspergillus fumigatus*, *Candida albicans*, *Cryptococcus neoformans* and *Histoplasma capsulatum* [33]–[37].

Despite the great number of evidences demonstrating the suppressive role of IL-10 and its detrimental effects to human and experimental fungal infections, direct studies investigating the consequences of IL-10 ablation in the immunity to PCM have not been performed so far. In the present work we studied the effects of IL-10-deficiency in the development of innate and adaptive immunity against *P. brasiliensis* using IL-10-deficient mice as experimental model. IL-10-deficient macrophages have increased phagocytic and fungicidal abilities associated with increased production of pro-inflammatory mediators. More importantly, compared with IL-10 sufficient mice, IL-10^−/−^ mice were found to develop more precocious T cell responses and efficiently control fungal loads without excessive tissue pathology. The regressive disease and decreased mortality rates of IL-10^−/−^ mice clearly demonstrate the detrimental effect of this cytokine to pulmonary PCM.

## Materials and Methods

### Ethics statement

Animal experiments were performed in strict accordance with the Brazilian Federal Law 11, 794 establishing procedures for the scientific use of animals, and the State Law establishing the Animal Protection Code of the State of São Paulo. All efforts were made to minimize suffering, and all animal procedures were approved by the Ethics Committee on Animal Experiments of the Institute of Biomedical Sciences of University of São Paulo (Proc.76/04/CEEA).

### Mouse strains

Breeding pairs of homozygous IL-10-deficient C57BL/6-IL10^tm1Cgn^ (IL-10^−/−^) and wild type (WT) control C57BL/6 mice (intermediate susceptibility to *P. brasiliensis*) were bred at the University of São Paulo animal facilities under specific-pathogen-free (SPF) conditions in closed-top cages. Male mice were 8 to 12 weeks of age at the time of infection, and clean food and water were given *ad libitum*.

### Fungus and mice infection

The highly virulent *P. brasiliensis* 18 isolate was used throughout this study. To ensure the maintenance of its virulence, the isolate was used after three serial animal passages [38]. *P. brasiliensis* 18 yeast cells were then maintained by weekly subcultivation in semisolid Fava Netto culture medium [39] at 36°C and used on the seventh day of culture. For infection studies, fungal cells were washed in PBS, counted in a hemocytometer and adjusted to 20×10^6^ cells ml^−1^. Individual cell counts were used after extensive elimination of clumped cells by spontaneous sedimentation, followed by buds disruption after repeated passage of fungal suspension by a tuberculin syringe connected to a hypodermic needle. The viability of fungal suspensions, determined by Janus Green B vital dye (Merk), was always higher than 80%. Mice were anesthetized and submitted to intra-tracheal (i.t.) *P. brasiliensis* infection as previously described [40]. Briefly, after intraperitoneal anesthesia, the animals were infected with 1×10^6^ *P. brasiliensis* 18 yeast cells, contained in 50 µL of PBS, by surgical i.t. inoculation, which allowed dispensing of the fungal cells directly into the lungs. The skin was then sutured, and the mice were allowed to recover under a heat lamp.

### Labeling of *P. brasiliensis* yeast cells

*P. brasiliensis* yeast cells were washed in phosphate-buffered saline (PBS; pH 7.2) and heat-killed at 60°C for 1 h. The yeast suspension was sonicated using 3 cycles of 10 s each (21% amplitude) with Sonics (Vibra Cell VCX 750; Sonics & Materials) to eliminate aggregates. Yeast cells were washed, adjusted to 1×10^6^ cells/ml in PBS, and then incubated with propidium iodide (PI; 100 g/ml; Sigma) for 30 min at 37°C. The yeast suspension was then washed three times with PBS and stored at 4°C until use.

### Phagocytic and killing assays

Thioglycollate-induced peritoneal macrophages were isolated by adherence (2 h at 37°C in 5% CO_2_) on plastic-bottom tissue-culture plates (1×10^6^ cells/well; 24 well-plate). Macrophages were washed to remove non-adherent cells and cultivated overnight with fresh complete medium (DMEM, Dulbecco's Modified Eagle's Medium, Sigma) containing 10% heat inactivated fetal calf serum, 100 U/ml penicillin and 100 µg/ml streptomycin) in the presence or absence of recombinant IFN-γ (20 ng/ml in culture medium, BD-Pharmingen). Macrophage cultures were then infected with PI-labeled *P. brasiliensis* yeast cells at a macrophage∶yeast ratio of 1∶1. The cells were co-cultivated for 2 h at 37°C in 5% CO_2_ to allow fungal adhesion and ingestion. Supernatants were aspirated and cells were washed with PBS to remove unbound yeasts. Macrophages were detached from plastic with fresh cold medium and a rubber cell scraper on ice. The cells were transferred to tubes, washed twice in PBS and centrifuged (400×*g*, 10 min, 4°C). The pellets were resuspended in 200 µL of PBS containing 1% FCS and immediately analyzed by flow cytometry on FACScalibur (Becton Dickinson). The granulocyte gates, as defined by size (forward scatter [FSC]) and granularity (side scatter [SSC]), were used to determine the macrophage population. Data were analyzed using the FlowJo software (Tree Star, Inc.). For fungicidal assays, macrophages were cultivated as previously described and infected with *P. brasiliensis* yeasts in a macrophage∶yeast ratio of 12.5∶1. After 48 h of culture, plates were centrifuged and supernatants removed. The wells were washed with distilled water to lyse macrophages, the suspensions collected and assayed for the presence of viable yeasts. All assays were done with five wells per condition in over three independent experiments.

### Colony forming units (CFU) assays

The number of viable microorganisms in infected organs (lung, liver and spleen) or macrophage lysates from experimental and control mice were determined by counting the number of CFU as previously described [41]. Briefly, 100 µL aliquots of serial dilutions from organ or macrophage suspensions were plated onto brain heart infusion agar (Difco) supplemented with 4% (v/v) horse serum (Instituto Butantan, São Paulo, Brazil) and 5% *P. brasiliensis* 192 culture filtrate, the latter constituting a source of growth-promoting factor. The plates were incubated at 35°C, and colonies were counted daily until no increase in counts was observed. The number (log_10_) of viable *P. brasiliensis* colonies per ml or gram of tissue was expressed as means ± standard errors (SEs).

### Measurement of nitric oxide (NO) production

Supernatants from macrophage cell cultures were separated and stored at −70°C. NO production was quantified by the accumulation of nitrite in the supernatants by a standard Griess reaction [42]. All determinations were performed in duplicate and expressed in µM NO.

### Mortality rates

Mortality studies were performed with groups of 12 IL-10^−/−^ and WT control mice inoculated i.t. with 1×10^6^ yeast cells or PBS. Deaths were registered daily for a 220-day period, and the median survival time post infection was calculated. Experiments were repeated twice.

### Delayed-type hypersensitivity (DTH assay)

The DTH reactions were evaluated just before sacrifice of same animals used in the CFU assays, by the footpad test according to previously determined conditions [43]. Briefly, mice were inoculated with 25 µl (5 µg) of Fava Nettos's antigen [44] and the footpad thickness was measured immediately before and 24 h after antigen inoculation. Uninfected mice submitted to the footpad test were used as controls.

### Histopathologic analysis

Histolopathogic studies were performed with IL-10^−/−^ and WT mice at week 8 after infection. Lungs, liver and spleen were collected, fixed in 10% formalin and embedded in paraffin. Five-micrometer sections were stained with hematoxilin-eosin (H&E) for analysis of the lesions and silver stained for fungal evaluation. Pathological changes were analyzed based on the size, morphology and cell composition of granulomatous lesions, presence of fungi and intensity of the inflammatory infiltrates. Morphometrical analysis was performed using a Nikon DXM 1200c digital camera (×10 magnification) and Nikon NIS Elements AR 2.30 software. The area of lesions (in µm^2^) was measured in 10 microscopic fields per slide (n = 4–6). Results were expressed as the mean ± standard error of the mean (SEM) for the total area of lesions for each animal.

### Lung leukocytes isolation

Lungs from each mouse were excised, washed in PBS, minced, and enzymatically digested for 1 hour in 15 mL of digestion buffer [RPMI, 5% fetal calf serum, 1 mg/mL collagenase and 30 µg/mL DNase (Sigma)]. After erythrocyte lysis using NH_4_Cl lysis buffer, cells were washed, resuspended in complete media, and centrifuged for 30 minutes at 2,000× *g* in presence of 20% Percoll (Sigma) to separate leukocytes from cell debris and epithelial cells [45]. Total lung leukocyte numbers were assessed in the presence of trypan blue using a hemocytometer; viability was always higher than 85%. The absolute number of a leukocyte subset was equal to the percentage of that cell subset multiplied by the total number of leukocytes recovered from the digested lung divided by 100.

### Measurement of serum *P. brasiliensis*-specific isotypes

Specific isotypes levels (total Ig, IgM, IgA, IgG1, IgG2a, IgG2b, and IgG3) were measured by a previously described ELISA employing a cell-free antigen [46] prepared by using a pool of different *P. brasiliensis* isolates (Pb339, Pb265 and Pb18). The average of the optical densities obtained with sera from control mice (PBS inoculated), 1∶20 diluted, was considered the cutoff for each respective isotype. Optical densities for each dilution of experimental sera were compared to the control values. The titer for each sample was expressed as the reciprocal of the highest dilution that presented an absorbance higher than the cutoff.

### Measurement of cytokines

Supernatants from lung homogenates or macrophage cultures were separated from cell debris by centrifugation at 2,000×*g* for 15 min, passed through 0.22 µm pore-size filters (Millipore), and stored at −70°C. Cytokines (TNF-α and IFN-γ) and MCP-1 levels from macrophage supernatants were measured by capture enzyme-linked immunosorbent assay (ELISA) with antibody pairs purchased from eBiosciences or BD Pharmingen. The ELISA procedure was performed according to the manufacturer's protocol and absorbances were measured with Versa Max Microplate Reader (Molecular Devices). Cytokine content from lung supernatants was assayed using the Cytometric Bead Array mouse Th1/Th2/Th17 cytokine CBA kit (BD Biosciences) following the manufacturer's instructions. Samples were analyzed using a BD FACSCanto flow cytometer and BD FACSDiva software. Data were formatted and further analyzed using BD CBA software.

### Flow cytometry analysis

For cell-surface staining, leukocytes were washed and resuspended at 1×10^6^ cells/mL in staining buffer (PBS, 2% fetal calf serum and 0.1% NaN_3_). Fc receptors were blocked by the addition of unlabeled anti-CD16/32 (Fc block; BD Pharmingen). The leukocytes were then stained in the dark for 20 min at 4°C with the optimal dilution of each antibody. Fluorescein isothiocyanate (FITC)-labeled anti-CD62L, alexa fluor 488 labeled anti-CD8 and anti-IA^b^, phycoerythrin (PE)-labeled anti-CD44, anti-CD11c, peridinin chlorophyll protein (PerCP)-labeled anti-CD4, anti-CD8, anti-F4/80, anti-CD11b monoclonal antibodies (BD Biosciences) were used. Cells were washed twice with staining buffer resuspended in 100 µl, and an equal volume of 1% paraformaldehyde (Sigma) was added to fix the cells. The stained cells were analyzed immediately using a FACScalibur equipment and Cell-Quest software (BD Biosciences), gating on macrophages or lymphocytes as judged from forward and side light scatter. Ten thousand cells were counted and the data expressed as the percentage or the absolute number of positive cells, which was calculated trough the percentage obtained by FACS and the number of cells determined in Neubauer chambers. For intracellular detection of FoxP3, leukocytes obtained from lung lesions were fixed and permeabilized using Cytofix/Cytoperm (BD Biosciences). Initially, cells were labeled with antibodies for cell surface molecules such as FITC-conjugated anti-CD4 and PE-conjugated anti-CD25. Next, the cells were fixed, permeabilized and stained with Cy 5,5-conjugated anti-FoxP3, for 30 min at 4°C. Cells were then washed twice with staining buffer, resuspended in 100 µl, and an equal volume of 2% formalin was added to fix the cells. A minimum of 20,000 events were acquired on FACScalibur flow cytometer (BD Biosciences) using the Cell-Quest software (BD Biosciences). The graphs represent the percentage of Foxp3^+^ cells in the gate of CD4^+^ CD25^+^ T cells.

### *Limulus* amoebocyte lysate activity assay

Solutions used for the preparation of yeast cell suspensions were tested for the presence of LPS using the *Limulus* amoebocyte lysate chromogenic assy (E-TOXATE, Sigma) and always showed LPS levels lower than 0.015 endotoxin unit (EU)/ml.

### Statistical analysis

All values are means ± SEM, unless otherwise indicated. Depending on the number of experimental groups, data were analyzed by Student's *t* test or two-way analysis of variance and the Bonferroni posttests to compare groups. Differences between survival times were determined with the LogRank test using GraphPad Prism software (GraphPad Software, San Diego, CA, USA). *P* value<0.05 was considered significant.

## Results

### IL-10 deficiency increases the phagocytic and fungicidal abilities of macrophages

**Figure 1:**
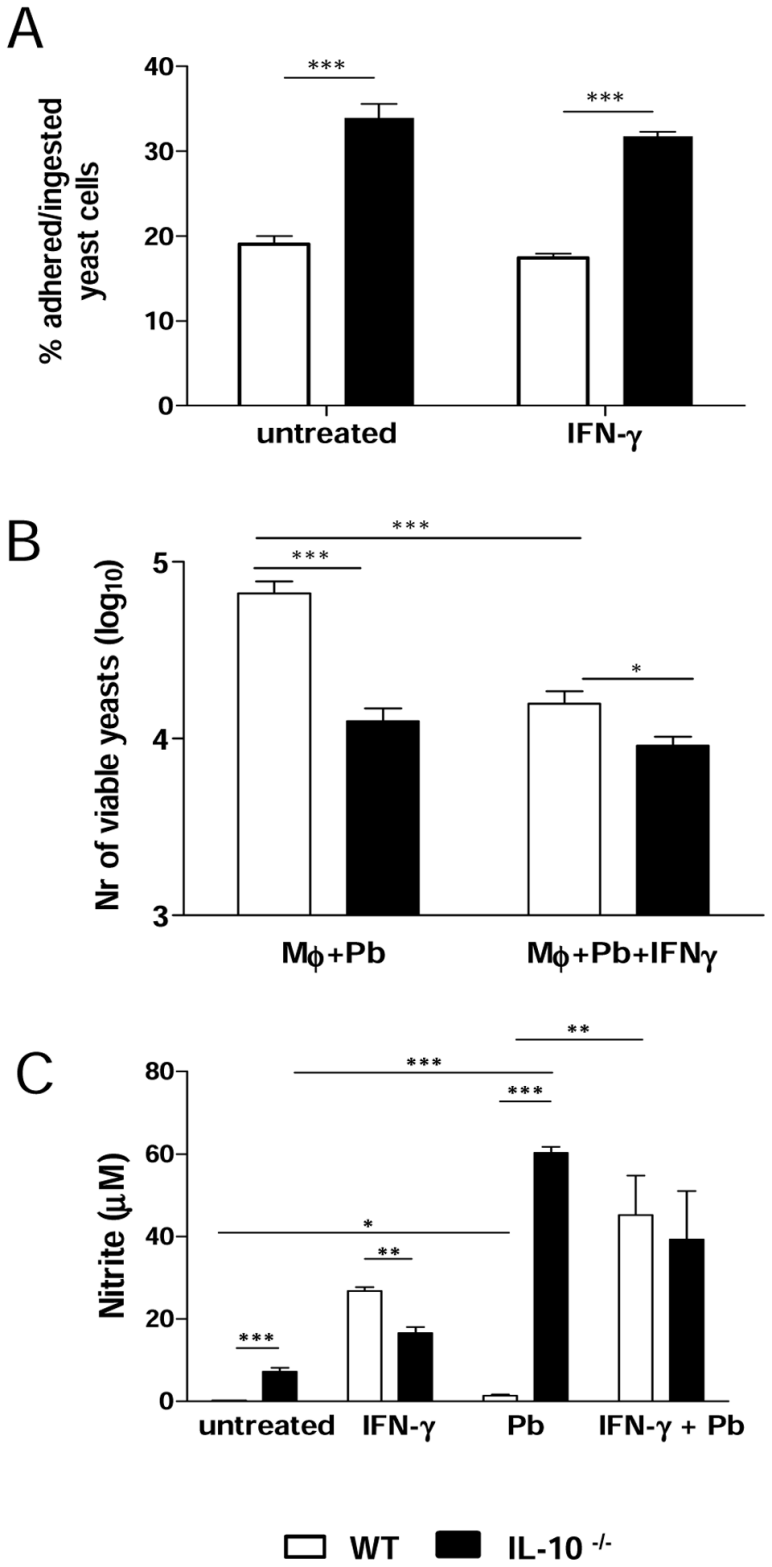
IL-10 inhibits the phagocytic and fungicidal abilities of macrophages. (A) Macrophages from WT and IL-10^−/−^ mice either treated with IFN-γ or left untreated were infected for 2 h with *P. brasiliensis* yeasts labeled with propidium iodide (1∶1, fungus∶macrophage ratio). Co-cultures were gently washed and macrophages were analyzed by flow cytometry. (B) For fungicidal assay, macrophages were infected with *P. brasiliensis* yeasts (1∶12.5, fungus∶macrophage ratio) during 2 h, washed, and further cultivated for 48 h at 37°C in 5% CO_2_. Supernatants were removed, the monolayers were washed with distilled water to lyse macrophages, and 100 µl of cell homogenates were assayed for the presence of viable yeasts by a CFU assay (C) NO production was measured in culture supernatants by Griess reagent. Data are means ± SEM of three independent experiments with similar results (* *p*<0.05, ** *p*<0.01 and *** *p*<0.001).

Firstly, we wanted to investigate whether the initial interactions between *P. brasiliensis* yeasts and peritoneal macrophages from WT and IL-10^−/−^ mice were equivalent. The phagocytic activity assay was carried out using PI-labeled *P. brasiliensis* yeast cells. Macrophages were previously treated with IFN-γ or left untreated and then cultivated with yeasts at a ratio of 1∶1. As shown in Figure 1A, macrophages from IL-10**^−/−^** mice displayed a significantly greater number of associated (ingested/adhered) yeasts than macrophages from WT mice. The same held true when macrophages were pre-treated with IFN-γ. The fungicidal activity of peritoneal macrophages was further determined after 48 h cultivation in the presence or absence of IFN-γ and lower numbers of viable yeast cells were recovered from deficient macrophages (Figure 1B). Interestingly, the pre-activation by IFN- γ increased the fungicidal activity of WT but not IL-10^−/−^ macrophages. Subsequently, supernatants from macrophages co-cultured with yeasts were assayed to detect the presence of NO. As seen in Figure 1C, in the absence of IFN-γ, higher levels of NO were produced by IL-10**^−/−^** macrophages relative to WT macrophages. When yeast cells were added, NO levels produced by IL-10^−/−^ cells increased dramatically. However, in the presence of IFN-γ, NO production was higher in the supernatants obtained from WT macrophages, but this difference was only significant in the absence of yeast cells. These results suggest that the lack of IL-10 increases macrophage function, such as phagocytic and fungicidal activities. With WT cells, the increased fungicidal activity could be correlated with increased NO production induced by IFN- γ treatment, whereas in IL-10^−/−^ macrophages a minor effect appeared to be mediated by the IFN- γ added, possibly by the intrinsic pre-activated state of IL-10-deficient cells.

### IL-10^−/−^ macrophages produce increased levels of IFN- γ, TNF-α and MCP-1

**Figure 2:**
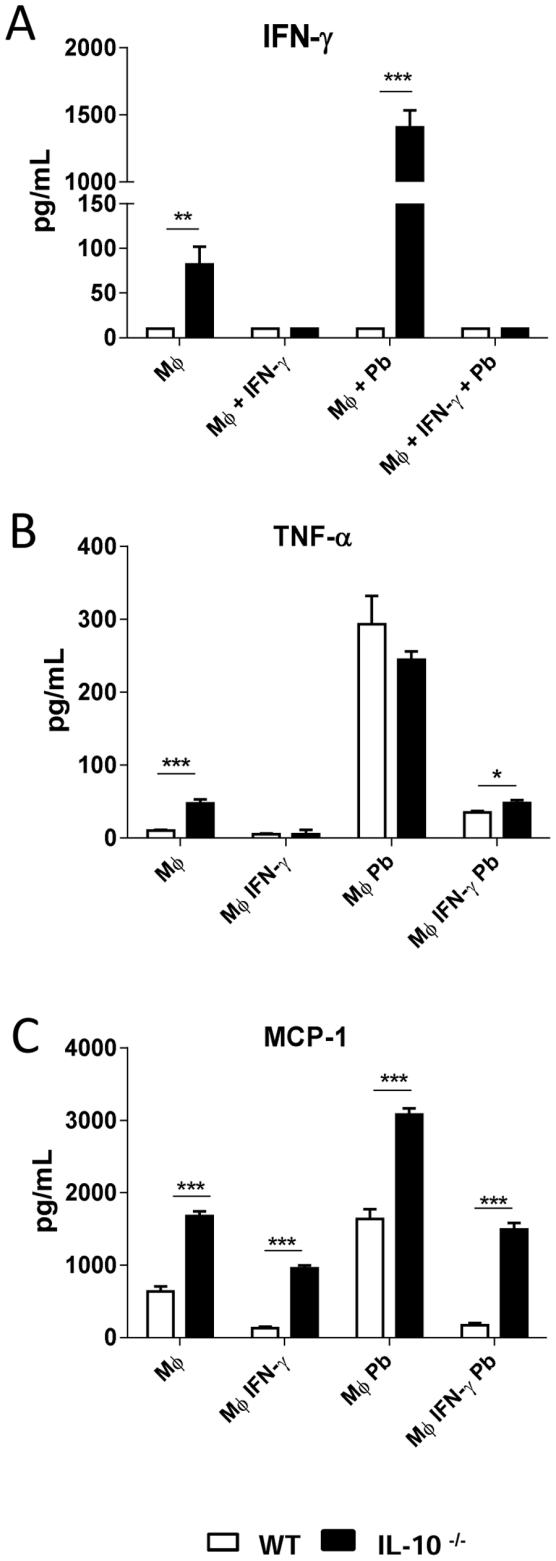
IL-10^−/−^ macrophages produce elevated levels of IFN- γ, TNF-α and MCP-1. Macrophages from WT and IL-10^−/−^ mice either treated with IFN-γ or left untreated were infected with *P. brasiliensis* yeasts (1∶25, fungus∶macrophage ratio) during 2 h, washed, and further cultivated for 48 h at 37°C in 5% CO_2_. Supernatants were removed and assayed for the presence of (A) IFN-γ (B) TNF-α, and (C) MCP-1. Data are means ± SEM of three independent experiments with similar results (* *p*<0.05, ** *p*<0.01 and *** *p*<0.001).

We have also evaluated the role played by *P. brasiliensis* infection in the production some pro-inflammatory cytokines (IFN- γ and TNF-α) and a chemokine (MCP-1). IFN-γ primed and unprimed macrophages were infected or not by *P.brasiliensis* and supernatants obtained after 48 h of cultivation. Compared with WT macrophages, uninfected and infected IL-10^−/−^ macrophages showed significantly increased IFN- γ levels. Priming by IFN- γ, however, abolished its own production (Figure 2A). Macrophages from IL-10-deficient mice also showed an augmented production of another pro-inflammatory cytokine, TNF-α (Figure 2B). Another important difference observed was the increased ability of IL-10^−/−^ macrophages to produce MCP-1, and this fact was consistent in all experimental groups analyzed (Figure 2C).

### IL-10^−/−^ mice show increased resistance to *P. brasiliensis* infection

**Figure 3:**
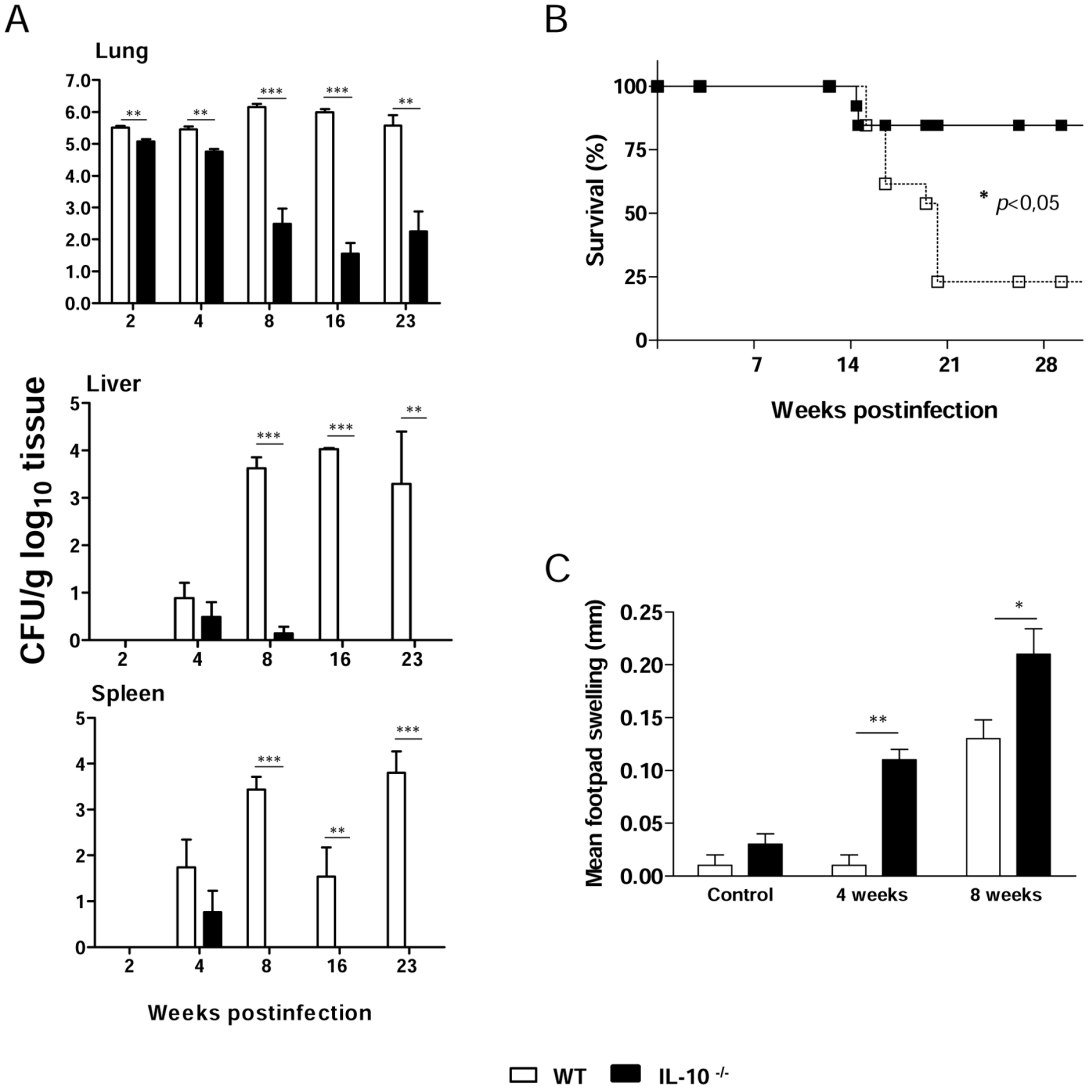
IL-10^−/−^ mice develop a less severe PCM associated with increased cellular immunity that results in decreased mortality rates. (A) IL-10^−/−^ and WT mice were inoculated with 1×10^6^ *P. brasiliensis* yeast cells by the i.t. route, and severity of infection was evaluated by determining fungal loads in the lungs, livers and spleens at five post-infection periods (2, 4, 8, 16 and 23 weeks). The bars depict means ± SEM of the numbers of log_10_ CFU obtained from groups of 6 to 8 mice. The results are representative of 3 experiments. ** *p*<0.01, and *** *p*<0.001, compared with WT controls. (B) Survival of IL-10^−/−^ and WT control mice after i.t. infection with 1×10^6^ *P. brasiliensis* yeast cells was determined for a period of 220 days. Results are representative of two independent experiments (n = 12; * *p*<0.05). (C) DTH responses mounted by *P. brasiliensis* infected mice. Control and infected WT and IL-10^−/−^ mice were injected intrafootpad with soluble *P. brasiliensis* yeast antigen (5 µg in 25 µl PBS) 24 hours before measurement of the footpad response at weeks 4 and 8 after fungal infection. Results are representative of two independent experiments. The bars depict means ± SEM of footpad swelling (* *p*<0.05; ** *p*<0.01; n = 6–8 mice).

In order to assess the role of IL-10 in the host defense against PCM, IL-10^−/−^ and WT mice were infected with virulent *P. brasiliensis* Pb 18 yeast cells and monitored for fungal load, mortality and DTH responses. Fungal burdens in the lungs, livers and spleens were determined after 2, 4, 8, 16 and 23 weeks of infection by CFU counting (Figure 3A). Mice genetically deficient in IL-10 were more resistant to systemic PCM than the WT controls, as demonstrated by the significantly reduced fungal load in lungs, livers and spleens. The number of lung CFU per gram of tissue was lower in IL-10^−/−^ mice at all time points analyzed, as compared to WT control mice (Figure 3A). Both mouse strains exhibited fungal dissemination to liver and spleen at week 4 post-infection. However, the CFU analysis revealed that IL-10^−/−^ mice were able to clear *P. brasiliensis* infection in livers and spleens after 16 and 8 weeks of infection, respectively (Figure 3A). Mortality of *P. brasiliensis*-infected WT and IL-10 KO mice was registered daily for a 220-day period. The survival curves of infected mice, depicted in Figure 3B, show significantly prolonged survival of IL-10^−/−^ mice compared to normal mice within the observation period.

To investigate the influence of IL-10 on DTH responses during PCM, mice were assessed for their ability to respond to Fava-Netto's antigen at weeks 4 and 8 after infection. Footpad swelling was analyzed 24 h after antigen inoculation in IL-10^−/−^ and WT mice. The results are shown in Figure 3C. Uninfected mice (negative controls) presented a nonspecific and small increase in footpad thickness. IL-10^−/−^ mice showed a significantly augmented reaction compared to normal mice at weeks 4 and 8 post-infection, suggesting a higher activation of the cellular immunity.

### IL-10^−/−^ mice develop reduced tissue pathology in the lungs and dissemination organs

**Figure 4:**
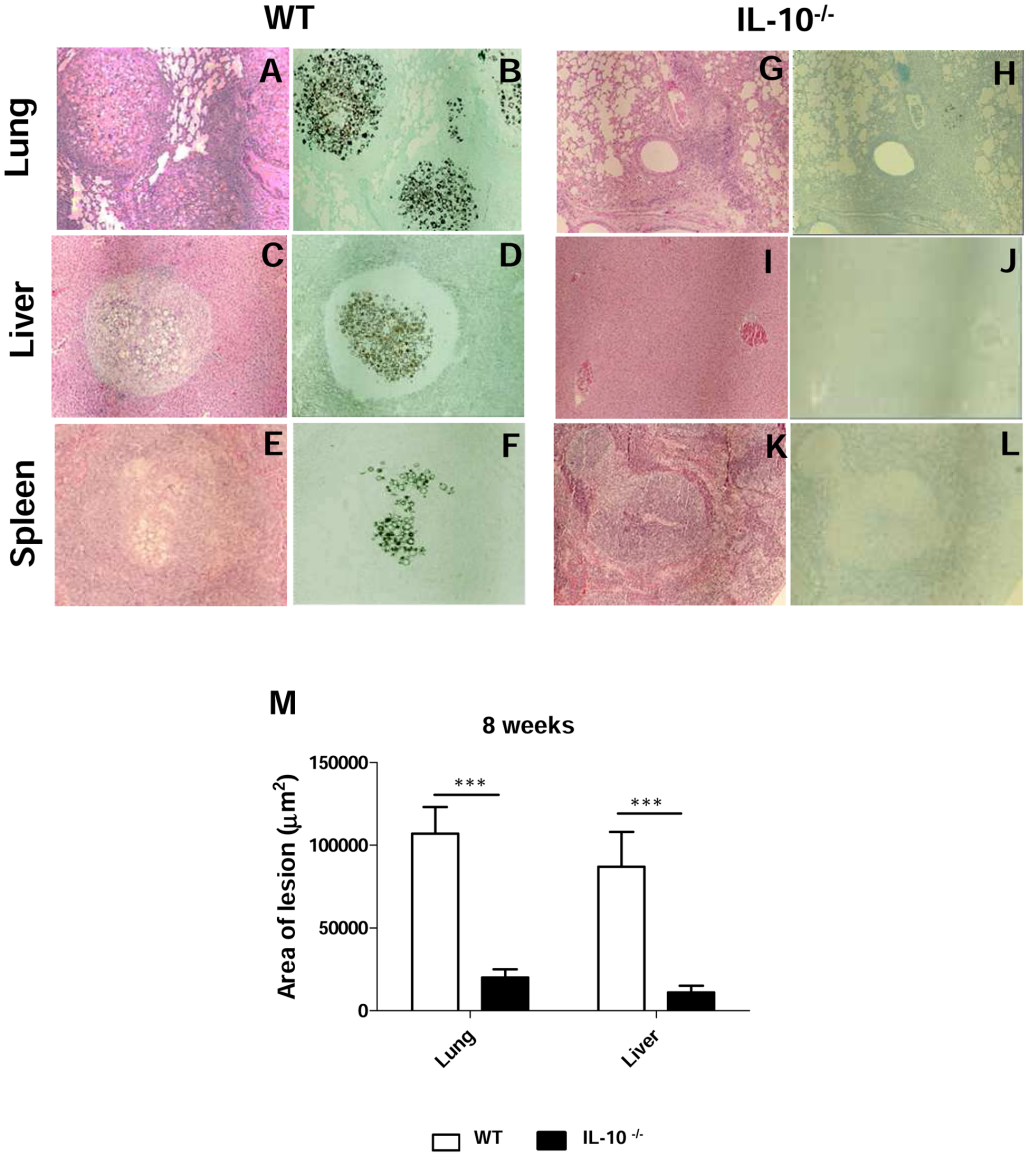
IL-10^−/−^ mice clear *P. brasiliensis* infection and develop mild pulmonary inflammatory reactions. Photomicrographs of lungs, livers and spleens from WT (A to F) and IL-10^−/−^ (G to L) mice at week 8 after infection with 1×10^6^ *P. brasiliensis* yeast cells. Compared with IL-10^−/−^ mice, increased number of inflammatory cells and more severe lesions were detected in WT mice. For panels: A, C, E, G, I and K, HE-stained, magnification ×10; for B, D, F, H, J and L, Groccot-stained, magnification ×10. Morphometrical analysis (M) confirmed the more extensive areas occupied by the lung and liver lesions of WT mice (*** *p*<0.001).

Histopathologic examination of organ sections from infected mice was performed at week 8 post-infection. As can be seen in Figure 4, the histologic appearance of the organs in *P. brasiliensis*-infected IL-10^−/−^ mice was considerably different from that in WT mice. Lung sections from all WT mice showed several confluent granulomatous lesions containing a high number of yeast cells, macrophages and polymorphonuclear cells, surrounded by a layer of lymphocytes (Figure 4A and B). By contrast, sections from IL-10^−/−^ mice mainly revealed the presence of resolved lesions, with few or no detectable yeast cells and better preservation of the pulmonary tissue. The inflammatory infiltrates present in restrict areas of parenchyma were mostly composed by macrophages and PMN neutrophils, with a reduced number of lymphocytes (Figure 4G and H). Livers and spleens from WT mice also showed foci of circumscribed granulomatous lesions and a peripheral sheet of lymphocytes (Figure 4C to F). These findings are in sharp contrast to those from IL-10-deficient mice, which presented normal liver and spleen morphologies (Figure 4I to L). Morphometric analyses of histological sections are depicted in Figure 4M. The areas of lesions in lungs and livers developed by WT mice were significantly larger than those developed by IL-10-deficient mice.

### In the course of infection, WT mice develop crescent levels of antibodies

Antibodies appear not to play a protective role in human and murine PCM although Th1/Th2-related isotypes have been used as good markers of the ongoing immune responses [30], [47]. In the severe forms of PCM, there is a predominance of Th2 isotypes (IgG1, IgG2b and IgA), whereas IgG2a, a Th1 isotype, is preferentially produced in the benign infection [30], [40].

**Figure 5:**
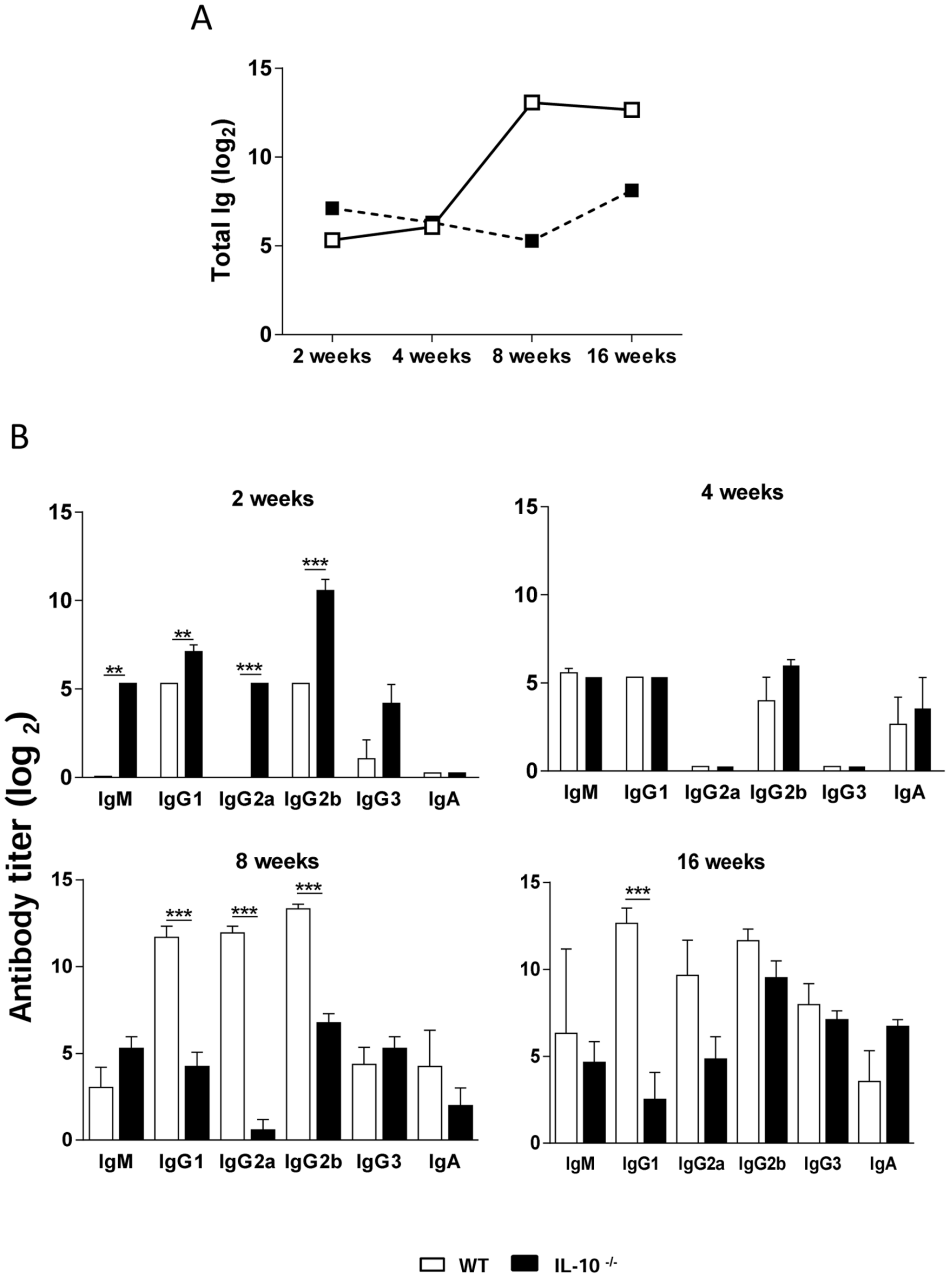
IL-10 deficiency determines increased humoral immunity in early *P. brasiliensis* infection. Levels of total specific Ig (A) and fungus-specific isotypes (B) in the sera of IL-10^−/−^ and WT mice at weeks 2, 4, 8, and 16 after i.t. infection with 10^6^ yeast cells. Sera were assayed for total Ig, IgM, IgA, IgG1, IgG2a, IgG2b and IgG3 by using an isotype-specific ELISA as detailed in *Materials and Methods*. The bars depict means ± serum titers (6 to 8 mice per group). The results are representative of 3 experiments. ** *p*<0.01; *** *p*<0.001, compared with WT controls.

When total specific Ig was measured in the course of infection, the early and elevated antibody response of IL-10^−/−^ mice, and the crescent antibody production of WT mice could be observed (Figure 5A). At week 2 post-infection, IL-10^−/−^ mice showed higher amounts of IgM as well as Th1- and Th2-related isotypes than WT mice. The latter produced undetectable amounts of IgG2a at this time point. By week 4, antibody titers were similar in both mouse strains, i.e., antibody production decayed in IL-10^−/−^ mice, whilst WT mice exhibited an increase in antibody synthesis. At weeks 8 and 16, WT mice showed higher antibody titers than IL-10-deficient mice (Figure 5B). Thus, resolution of *P. brasiliensis* infection might be associated, among other factors, with an early activation of humoral immunity and production of Th1-related isotypes.

### The late production of cytokines is associated with susceptibility to *P. brasiliensis* infection

**Figure 6:**
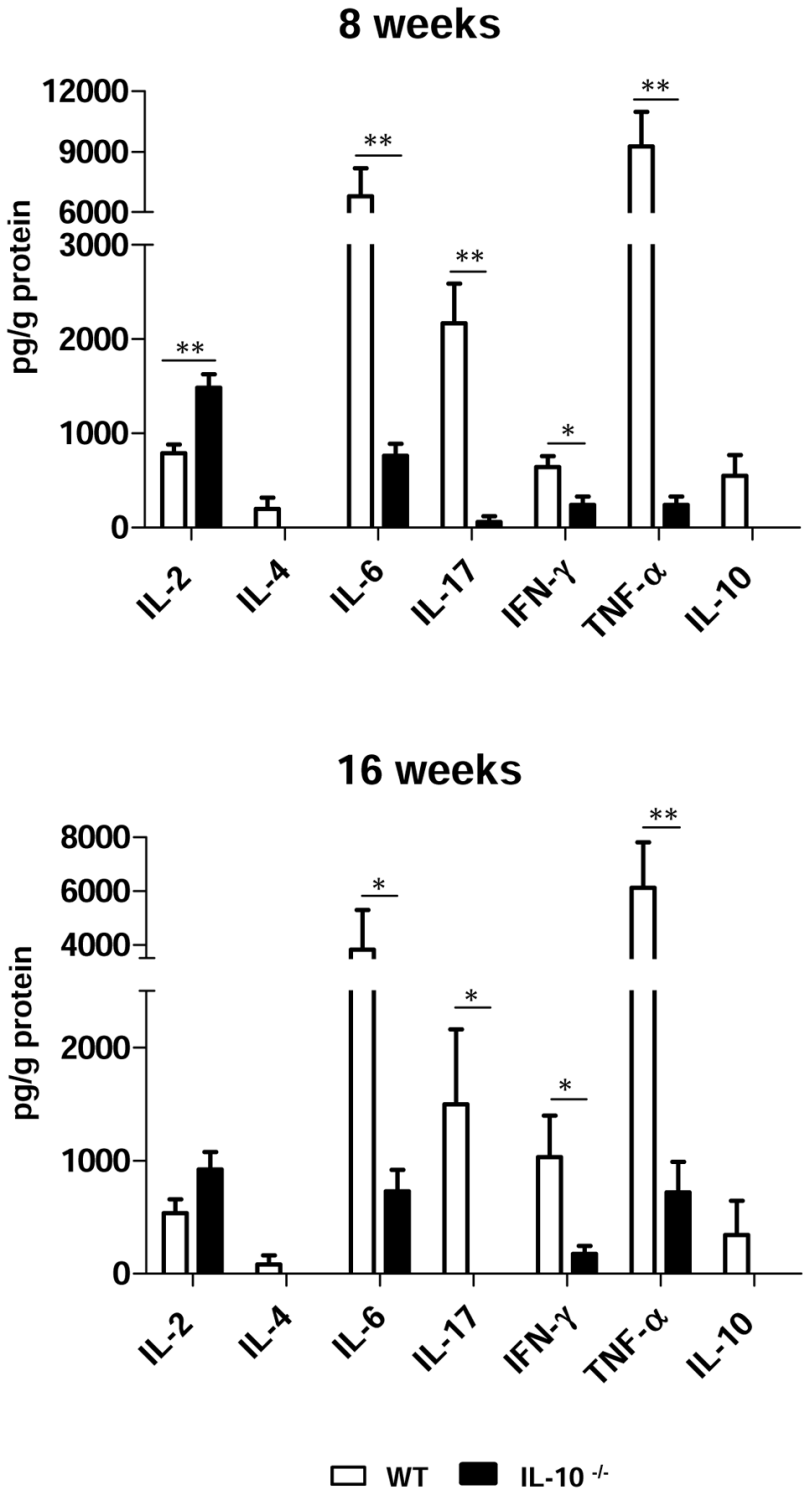
At late stages of infection, IL-10 deficiency leads to reduced production of pro- and anti-inflammatory cytokines in the lungs of *P. brasiliensis*-infected mice. Levels of cytokines in lung homogenates of IL-10^−/−^ and WT control mice were measured after i.t. infection with 10^6^ yeast cells. Lungs were disrupted at weeks 8 and 16 after infection, and supernatants were analyzed for cytokine content by using the BD CBA mouse Th1/Th2/Th17 cytokine kit. The bars depict means ± SEM of cytokine levels (6 to 8 animals per group). * *p*<0.05; ** *p*<0.01, compared with WT control.

It has been repeatedly shown that the proinflammatory cytokines IFN-γ, TNF-α and IL-12 are linked to disease protection, whilst anti-inflammatory cytokines, such as IL-4, IL-10 and TGF-β, prevail in severe forms of PCM [30], [48], [49]. Th17-related cytokines, in turn, have been demonstrated to exert opposite effects on PCM: they enhance the number and effector activity of neutrophils but also lead to augmented inflammatory reaction mixtures [4]. Herein, the levels of Th1/Th2/Th17 cytokines in lung homogenates of IL-10^−/−^ and WT mice were determined at weeks 8 and 16 after *P. brasiliensis* infection (Figure 6). Production of IL-2 was higher in IL-10^−/−^ mice than in their WT counterparts at both time intervals. IL-4 could only be detected in lung homogenates from IL-10^−/−^ mice, albeit at very low levels. At both time points analyzed, lower levels of IL-5, IL-17, IFN-γ and TNF-α were present in lung homogenates from IL-10^−/−^ mice than WT mice, which also showed production of IL-10. These results demonstrate that the reduced levels of cytokines observed in IL-10^−/−^ mice reflect their increased ability of pathogen clearance.

### Resistance of IL-10^−/−^ mice to *P. brasilensis* is associated with an early activation of cell-mediated immunity

**Figure 7:**
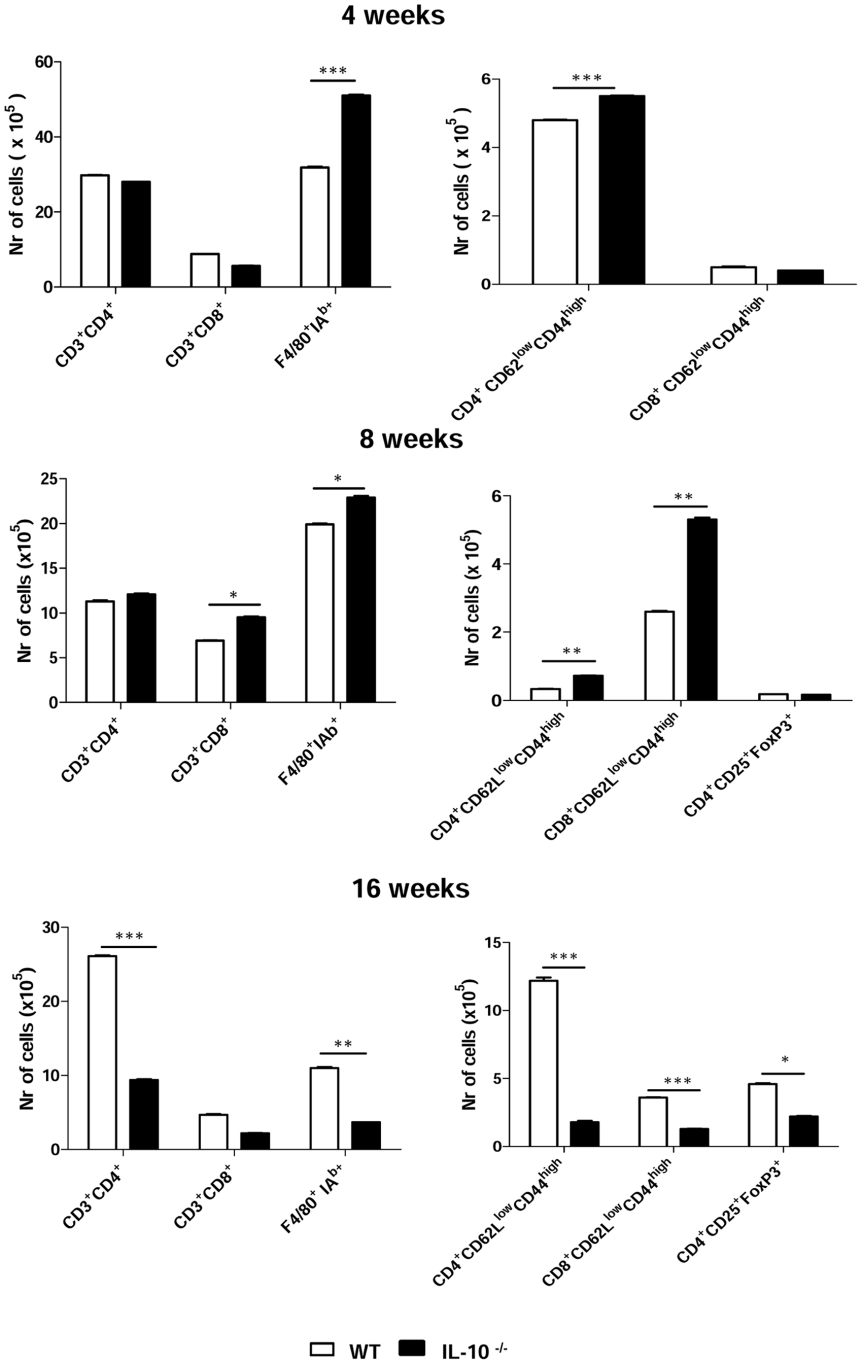
Resistance of IL-10^−/−^ mice to *P brasiliensis* infection is associated with upregulation of cellular immunity. Characterization of leukocyte subsets and the activation profile of lymphocytes by flow cytometry in the lungs from IL-10^−/−^ and WT mice inoculated with 1×10^6^ *P. brasiliensis* yeast cells. At week 4, 8 and 16 after infection, lung suspensions were obtained and stained as described in Materials and Methods. To characterize the expansion of Treg cells, lung homogenates from IL-10^−/−^ and WT mice were stained for surface CD4 and CD25 and intracellular FoxP3 expression. The data represent the mean ± SEM of the results from 6 to 8 mice per group and are representative of two experiments (* *p*<0.05; ** *p*<0.01, *** *p*<0.001).

Next, the inflammatory infiltrates in the lungs of infected mice were characterized. The presence of monocytes/macrophages (F4/80^+^IA^b+^), T cells (CD4^+^ or CD8^+^), as well as the activation status of T cells were determined at weeks 4, 8 and 16 after *P. brasiliensis* infection. Figure 7 shows that, although the number of CD4^+^ and CD8^+^ T cells were similar in both mouse strains at week 4, the amount of activated CD4^+^ T cells (cells expressing low levels of CD62L and high levels of CD44 [50]) was significantly higher in IL-10^−/−^ mice. In addition, an augmented number of phagocytes were also detected in IL-10^−/−^. By week 8, the amount of CD4^+^ T cells in the lungs of both WT and IL-10^−/−^ mice was still equivalent, while the number of CD8^+^ T cells and phagocytes, as well as of activated CD4^+^ and CD8^+^ T lymphocytes were significantly augmented in IL-10^−/−^ mice compared to their normal counterparts, indicating that early in the infection activated T cells migrated to the lungs of IL-10-deficient mice. Interestingly, while the total number of activated CD4^+^ cells decreased at this time point, the number of CD8^+^ T cells increased. Later in the course of infection, at week 16, all cell populations analyzed were found in higher numbers in WT mice than in IL-10^−/−^ mice. This difference was significant for CD4^+^ T cells and macrophages, but not for CD8^+^ T cells. However, increased numbers of activated CD8^+^ T cells were detected at this period of infection.

To rule out the possibility that the diminished immune responses presented by IL-10-deficient mice late in *P. brasiliensis* infection could have occurred due to the action of regulatory T (Treg) cells, this cell population was quantified in lung homogenates of IL-10^−/−^ and WT mice at weeks 8 and 16 after infection (Figure 7). Compared to WT mice, lower numbers of CD4^+^CD25^+^FoxP3^+^ Treg cells were detected in lungs of IL-10^−/−^ mice at the 16^th^ week post-infection. This difference was significant (p<0.05), demonstrating that the reduced immune responses presented by IL-10-deficient mice in later stages of infection are not associated with the presence of Treg cells. Rather, they underscore our findings demonstrating that IL-10^−/−^ mice are able to resolve an infection with *P. brasiliensis*. Additionally, these findings suggest that the presence of lower expansion of Treg cells may be associated with an increased efficiency of effector T cells from IL-10^−/−^ mice.

## Discussion

The deleterious role of IL-10 in diverse models of infection with pathogenic fungi has been intensively studied [34], [35], [37]. However, the *in vivo* effects of IL-10 deficiency throughout *P. brasiliensis* infection have not been previously assessed. In the present work, we sought to investigate whether the absence of functional IL-10 impacted the course of systemic PCM. Firstly, our results showed that IL-10 deficiency is associated with increased macrophage effector functions, such as phagocytic and fungicidal activities as well as NO synthesis. This fact is consistent with observations for other fungal pathogens, such as *C. albicans* [35]. The suppressive role of IL-10 on phagocyte killing activity is well established [31], [32], [51]. Furthermore, IL-10-induced suppression of phagocyte antimicrobial activity is often linked to the down-regulated production of inflammatory cytokines, such as TNF-α and IFN-γ [31], [52]. The mechanisms of macrophage activation by IFN-γ as well as the contrasting effects of IFN-γ and IL-10 on phagocyte functions have been repeatedly demonstrated [32], [53], [54]. The elevated spontaneous- and *P. brasiliensis*-induced production of TNF-α, IFN-γ, MCP-1 and NO here observed appears to explain the increased phagocytic and fungicidal activities of IL-10-deficient macrophages and their refractoriness to IFN- γ priming. These findings suggest that macrophages derived from IL-10-deficient mice were already primed due to the lack of the inhibitory activities of IL-10, so that addition of exogenous IFN-γ did not cause any significant increase in NO and cytokines production. It can also be hypothesized that these cells display a prevalent M1 pro-inflammatory phenotype expressing high levels of SOCS (suppressor of cytokine signaling) proteins, which would explain the reduced macrophage response to IFN- γ activation [54], [55]. However, the challenge by fungal cells appears to provide the second signal needed to the efficient activation of inducible NO-synthase (iNOS). This response was possibly mediated by the direct interaction of fungal cells with pathogen recognition receptors on macrophage membrane and IFN-γ-independent cell signaling (55). Our results have also indicated that IL-10^−/−^ macrophages use NO-independent mechanisms to control fungal growth. Therefore, the production of microbicidal peptides and enzymes, the depletion of endogenous tryptophan by 2,3 indoleamine dyoxigenase induced by IFN-γ-independent mediators, the production of eicosanoid mediators and reactive oxygen intermediates [1] could also be involved in the increased fungicidal ability of IL-10 deficient macrophages. In addition, the elevated production of MCP-1 by IL-10^−/−^ macrophages can be correlated with the increased production of pro-inflammatory cytokines and the efficient control of fungal growth displayed by these cells. Indeed, in murine cryptococcosis MCP-1 was shown to control the early production of pro-inflammatory cytokines such as TNF-α and IL-6, the recruitment of mononuclear cells to infected lungs and the immunoprotection mediated by Th1 immunity [56], [57].

IL-10 deficiency resulted in lower fungal burdens in lungs and diminished dissemination to other target organs. IL-10^−/−^ mice were able to clear *P. brasiliensis* in liver and spleen and showed reduced tissue injury, as well as greater survival times after infection. In this way, absence of IL-10 was beneficial in our murine model of PCM, since the fungal burden was significantly reduced without concomitant tissue damage, leading to dramatically diminished mortality rates. This more efficient control of infection by IL-10-deficient mice is consistent with reports for other clinically relevant pathogens including bacteria such as *Mycobacterium tuberculosis* and *Chlamydia tracomatis* [58], [59]; protozoa such as *Leishmania major* and *Leishmania donovani* [60], [61]; and fungi such as *C. albicans*, *C. neoformans* and *H. capsulatum* [37], [34], [35]. Nevertheless, our results contrast with those from other groups, who showed that IL-10 prevented mice of succumbing to some experimental infections, such as those with *Plasmodium chabaudi chabaudi*, *Trypanosoma cruzi* and *Toxoplasma gondii* [62]–[64]. In these cases, however, IL-10 conferred protection by inhibiting the development of excessive immune activation and exaggerated inflammatory responses [reviewed by 65]. Therefore, since IL-10 has been demonstrated to be a pleiotropic cytokine with a great variety of effects on the immune system [reviewed by 17], the beneficial or detrimental effects of its broad anti-inflammatory activities seem to depend on the pathogen involved.

Our findings on tissue pathology and survival are in line with the augmented cellular immune responses that led to increased DTH reactions in IL-10-deficient mice. Cell-mediated immunity that results in positive DTH reactivity and acquired resistance to infection is mainly orchestrated by Th1 cells [66], [67]. Stronger DTH responses in IL-10^−/−^ mice have been demonstrated for *Chlamydia trachomatis* [59]. Furthermore, we provided evidence that IL-10-deficient mice showed early production of Th1-related antibodies, predicting the onset of a less severe infection. This outcome is consistent with previous findings from our group [30]. The mechanisms by which specific antibodies induce protection against fungi are not well elucidated [68], but it is known that antibodies may impact the Th1/Th2-type cytokine balance and the induction of regulatory T cells [reviewed by 2]. The presence of a Th1-polarizing isotype in IL-10-deficient mice early in the course of infection may have contributed to disease resolution, in agreement with previous findings for infections with some pathogenic fungi, such as *A. fumigatus* and *C. albicans*, or intracellular bacteria, such as *M. tuberculosis* and *L. monocytogenes* [36], [69]–[71]. However, early in infection (2 weeks), IL-10-deficient mice showed significantly higher titers of IgG1, IgG2a, and IgG2b, suggesting the development of mixed Th1/Th2 responses. The higher antibody levels, together with the lower fungal burden in the initial steps of disease might indicate a precocious activation of immunity in IL-10^−/−^ mice. Guimarães et al. [72] have demonstrated that IgG1 and IgG2a antibodies against a surface protein of *H. capsulatum* reduced fungal burden and decreased inflammation, in part by polarizing to a Th1 response. Interestingly, we also detected higher IgM titers in IL-10^−/−^ mice at the beginning of infection, which may also have limited disease progression, since it has been shown that IgM antibodies against a surface protein of *H. capsulatum* led to augmented phagocytosis and killing of this fungal pathogen by macrophages, diminished pulmonary inflammation and prolonged the survival of lethally infected mice [73].

In late stages of infection, we observed higher amounts of cytokines in lung homogenates from WT mice, as compared to IL-10^−/−^ mice, except for IL-2. At weeks 8 and 16, IL-10-deficient mice had nearly cleared infection, suggesting that cytokine production was no longer required. The same held true in relation to the number of macrophages and activated/memory T cells in the lungs of infected mice. IL-10-deficient mice exhibited an augmented migration of these cell types in the beginning of infection, whereas at later time points the presence of these cell populations was more pronounced in IL-10-sufficient mice. In a murine model of histoplasmosis, Deepe and Gibbons [37] demonstrated that the faster pathogen clearance in IL-10^−/−^ mice was due to an increased biological activity of memory T lymphocytes from those mice, rather than higher cell numbers at some post-infection periods.

Some lines of investigation showed that production of IL-10 was associated with the generation of Treg cells in mice infected with *C. albicans* [74], [75]. In keeping with these findings, we found lower numbers of Treg cells in IL-10-knockout mice. The deleterious effects of Treg cells on PCM have been recently shown by our group [5], so that a better disease outcome when Treg are kept in lower levels was not unexpected.

In summary, our results clearly demonstrate that the absence of IL-10 leads to beneficial effects in pulmonary PCM, since it allows disease control without causing significant tissue damage consequent of overproduction of pro-inflammatory cytokines. Thus, our data suggest that modulation of IL-10 production could be an important immunotherapy approach for PCM.
